# Competing risks modeling of length of hospital stay enhances risk-stratification of patient care: application to under-five children hospitalized in Malawi

**DOI:** 10.3389/fepid.2023.1274776

**Published:** 2023-11-13

**Authors:** Christopher C. Stanley, Madalitso Zulu, Harrison Msuku, Vincent S. Phiri, Lawrence N. Kazembe, Jobiba Chinkhumba, Tisungane Mvalo, Don P. Mathanga

**Affiliations:** ^1^ MAC-Communicable Diseases Action Centre, Kamuzu University of Health Sciences, Blantyre, Malawi; ^2^ School of Global and Public Health, Kamuzu University of Health Sciences, Blantyre, Malawi; ^3^ University of North Carolina Project Malawi, Lilongwe, Malawi; ^4^ Department of Computing, Mathematical and Statistical Sciences, University of Namibia, Windhoek, Namibia; ^5^ Department of Pediatrics, School of Medicine, University of North Carolina at Chapel Hill, Chapel Hill, NC, United States

**Keywords:** competing risks, Kaplan-Meier curve, Cox proportional hazards, modeling, hospital stay, Malawi

## Abstract

**Introduction:**

Length of hospital stay (LOS), defined as the time from inpatient admission to discharge, death, referral, or abscondment, is one of the key indicators of quality in patient care. Reduced LOS lowers health care expenditure and minimizes the chance of in-hospital acquired infections. Conventional methods for estimating LOS such as the Kaplan-Meier survival curve and the Cox proportional hazards regression for time to discharge cannot account for competing risks such as death, referral, and abscondment. This study applied competing risk methods to investigate factors important for risk-stratifying patients based on LOS in order to enhance patient care.

**Methods:**

This study analyzed data from ongoing safety surveillance of the malaria vaccine implementation program in Malawi's four district hospitals of Balaka, Machinga, Mchinji, and Ntchisi. Children aged 1–59 months who were hospitalized (spending at least one night in hospital) with a medical illness were consecutively enrolled between 1 November 2019 and 31 July 2021. Sub-distribution-hazard (SDH) ratios for the cumulative incidence of discharge were estimated using the Fine-Gray competing risk model.

**Results:**

Among the 15,463 children hospitalized, 8,607 (55.7%) were male and 6,856 (44.3%) were female. The median age was 22 months [interquartile range (IQR): 12–33 months]. The cumulative incidence of discharge was 40% lower among HIV-positive children compared to HIV-negative (sub-distribution-hazard ratio [SDHR]: 0.60; [95% CI: 0.46–0.76]; *P* < 0.001); lower among children with severe and cerebral malaria [SDHR: 0.94; (95% CI: 0.86–0.97); *P* = 0.04], sepsis or septicemia [SDHR: 0.90; (95% CI: 0.82–0.98); *P* = 0.027], severe anemia related to malaria [SDHR: 0.54; (95% CI: 0.48–0.61); *P* < 0.001], and meningitis [SDHR: 0.18; (95% CI: 0.09–0.37); *P* < 0.001] when compared to non-severe malaria; and also 39% lower among malnourished children compared to those that were well-nourished [SDHR: 0.61; (95% CI: 0.55–0.68); *P* < 0.001].

**Conclusions:**

This study applied the Fine-Gray competing risk approach to more accurately model LOS as the time to discharge when there were significant rates of in-hospital mortality, referrals, and abscondment. Patient care can be enhanced by risk-stratifying by LOS based on children's age, HIV status, diagnosis, and nutritional status.

## Introduction

Length of hospital stay (LOS), defined as the time from inpatient admission to discharge, death, referral, or abscondment, is one of the key indicators of quality in patient care (1). Apart from infrastructure, LOS is another metric recommended by the World Health Organization (WHO) to measure health service quality and efficiency (2). LOS has direct implications on costs and resources for care at all levels of a health system (household, hospital, community, government, and global), as shorter LOS is associated with reduced resource consumption, resulting in lower health-related expenditures (3, 4). Furthermore, shorter LOS minimizes the chance of in-hospital acquired infections, resulting in improved quality of care and treatment outcomes and reduced morbidity and mortality (5).

The common methods for estimating time to discharge include the Kaplan-Meier (K-M) estimator and Cox proportional hazards regression. The cumulative incidence is usually estimated using the K-M complement of the survival curve (i.e., 1-KM) while cause-specific hazards (CSH) are usually estimated using the Cox proportional hazards regression (6, 7). The underlying assumption in these methods is that the probability of the outcome (in this case discharge) is the same among individuals still under observation and those censored (8–10) which may not always be true in real-world situations. Specifically, with the conventional approaches, deaths, referrals, and absconders are censored as in a standard survival analysis (11, 12). Therefore, both of these methods ignore the influence that time to death, referral, or abscondment have on the cumulative incidence of discharge. In real situations, discharge is no longer possible (i.e., is precluded) when an individual dies, absconds, or is referred to another hospital. Appropriate analytical methods for time-to-event data in the presence of competing events are competing risk survival methods such as the Fine-Gray model (12, 13). Determining factors that may lower LOS among children under 5 years may help streamline resource allocation and customize appropriate interventions.

In Malawi, common diseases for hospitalization among children under 5 years include malaria (inclusive of febrile, severe, and cerebral), pneumonia, diarrhea, sepsis, and meningitis (14, 15). There is a need to explore options that optimize the use of available scarce health resources by risk-stratifying patients based on LOS where possible in order to enhance patient care. This study applies the Fine-Gray competing risk model to investigate factors important for risk-stratifying patients based on LOS in order to enhance patient care.

## Methods

### Data

**Figure 1:**
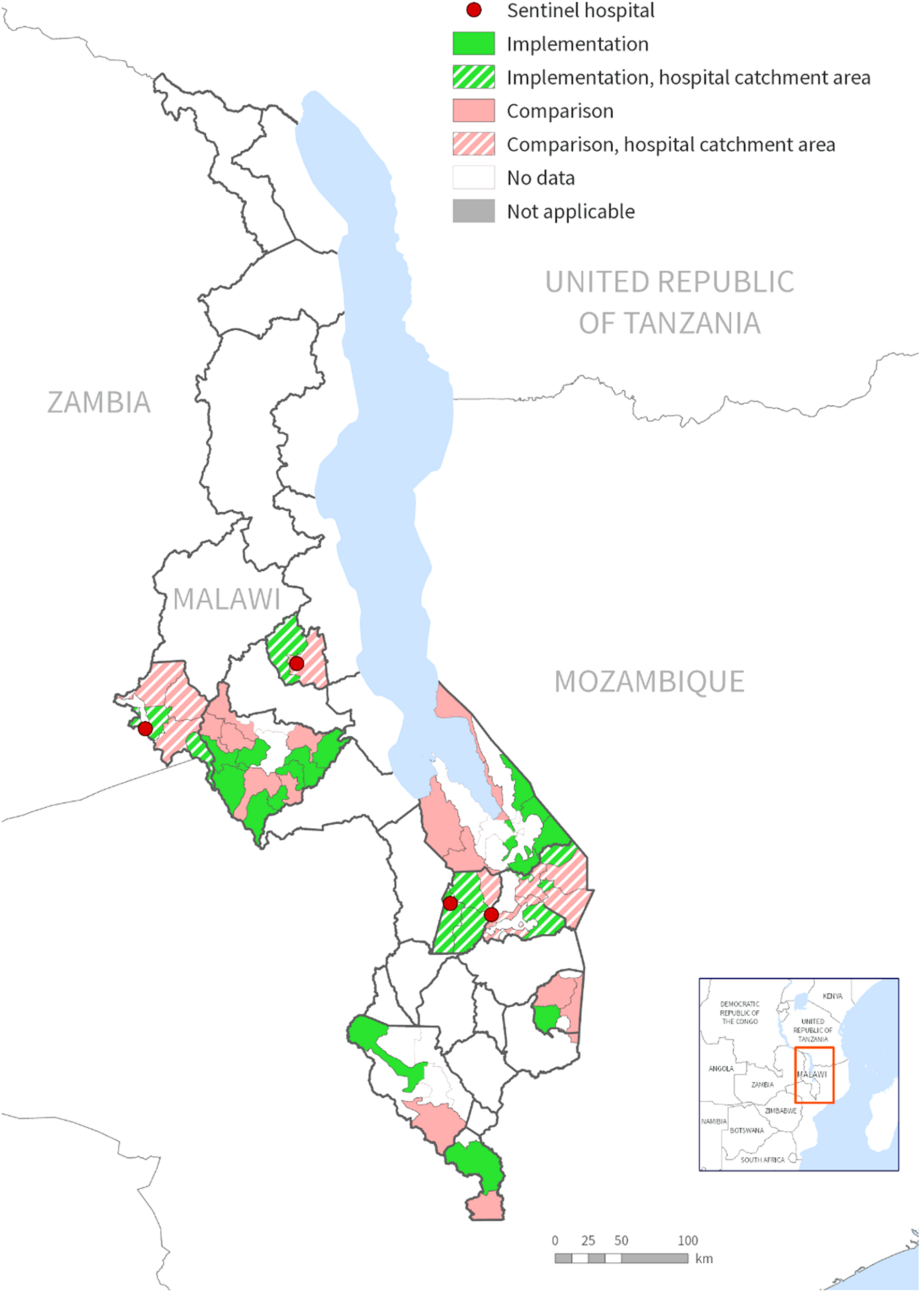
Map of Malawi showing districts implementing the malaria vaccine implementation program (MVIP) and sentinel hospital sites for the safety surveillance.

This study analyzed data from ongoing safety surveillance of the malaria vaccine implementation program (MVIP) in Malawi. The MVIP is a cluster randomized evaluation of the introduction of the RTS, S/AS01 malaria vaccine being conducted in Malawi, Kenya, and Ghana (Clinical Trials Number NCT03806465). A detailed description of the Malawi MVIP is already published elsewhere (16). In Malawi, the MVIP evaluation is being conducted in nine districts that are piloting the malaria vaccine implementation. The districts are spread across the central (Ntchisi, Lilongwe rural, and Mchinji) and southern (Balaka, Mangochi, Machinga, Phalombe, Chikwawa, and Nsanje) regions of Malawi (Figure 1). In all the MVIP districts, malaria transmission is intense and year-round, peaking during the rainy season (November-May). *Plasmodium falciparum* is the dominant parasite species although *Plasmodium malariae* and *Plasmodium ovale* have been recorded (17). Hospital surveillance is limited to only four district hospitals that serve as sentinel sites for the safety surveillance module. These hospitals are Balaka District Hospital (BDH), Machinga District Hospital (MDH), Mchinji District Hospital (MCDH), and Ntchisi District Hospital (NDH), all of which operate as secondary-level hospitals. Balaka and Machinga district hospitals are located in the southern region of Malawi, serving a population of 450,000 and 500,000 respectively. Mchinji and Ntchisi district hospitals are in the central region of Malawi and have a population of 630,000 and 320,000 respectively. The pediatric admissions to these hospitals range from 2,500–5,000 children annually. The four districts were chosen because of their high prevalence of severe malaria cases and the existence of basic laboratory infrastructure necessary to support the hospital surveillance. Each hospital serves as a referral center for primary health facilities and public or private clinics across the entire district.

### Inclusion and exclusion criteria

Children aged 1–59 months who were hospitalized (spending at least one night at the selected district hospitals) in the wards with a medical illness were consecutively enrolled in the surveillance if their guardians provided verbal consent. Children were recruited in the study irrespective of whether they received the RTS, S/AS01 malaria vaccine or not. Children were excluded from the study if guardians did not provide consent to be part of the surveillance. This analysis included data from 15,463 children who were hospitalized between 1 November 2019 and 31 July 202.

### Outcome

The primary outcome of this study was the length of hospital stay (LOS) defined as time to discharge calculated as time in days from the date of admission.

### Covariates

The covariates included child demographics (age and gender), vital signs (weight, temperature), oxygen saturation, nutritional status defined as malnourished if mid-upper arm circumference (MUAC) < 11.5 cm, Blantyre coma score (BCS) for the level of consciousness ranging from 0 to 5 with 0 indicating a poor result and 5 indicating a good result, severe anemia (hemoglobin < 5 g/dl), history of fever in the past week (yes/no), primary final diagnosis (malaria, pneumonia, sepsis, meningitis, or other diseases), and HIV status (negative, positive, exposed to infected mother, or unknown).

### Data analysis

Baseline characteristics were summarized using numbers with percentages if categorical or medians with interquartile ranges (IQRs) if continuous but skewed. Cumulative incidence, as opposed to event rate alone, is recommended for informing healthcare planning and decision-making because it describes the trend of events over time (18, 19). Cumulative incidences were estimated from a competing risk model and compared to the conventional method of using the complement of the K-M curve. Sub-distribution-hazard (SDH) ratios were estimated with the Fine-Gray model in order to assess associations with the cumulative incidence of discharge while accounting for the competing events of death, referral, and abscondment (20). The Fine-Gray model uses a sub-distribution hazard to model the effect of covariates on the cumulative incidence (12, 13). The rate of discharge was estimated as a cause-specific hazard (CSH) using Cox proportional hazards regression (10, 12, 21). Confidence intervals (CIs) were reported as 95%, and the threshold for significance was considered at a two-sided alpha level of 0.05. All analyses were done using Stata SE version 15.1 (Stata Corp., College Station, TX) (22).

## Results

Among the 15,463 children hospitalized in this study between 1 November 2019 and 31 July 2021 in the four hospitals, 8,607 (55.7%) were male and 6,856 (44.3%) were female (Table 1). The median age was 22 months [interquartile range (IQR): 12–33 months], with 3,526 (222.8%) children aged 1–11 months, 8,855 (57.3%) 12–35 months, and 714 (20.9%) 36–59 months. On admission, 5,814 (52.2%) out of 11,144 children with a documented temperature had a fever (i.e., >37.5°C), although 12,924 (83.6%) reported a history of fever in the 7 days prior to being hospitalized. Out of the 15,463 children hospitalized, 15,355 (99.3%) children had a documented HIV status of which 7, 654 (49.8%) were HIV-negative, 89 (0.6%) were HIV-positive, 147 (1.0%) were exposed to HIV-infected mothers but not infected, and 7,465 (48.6%) were unknown. All 15,463 children had a measurement of the mid-upper arm circumference (MUAC) of which 936 (6.1%) were malnourished (MUAC < 11.5 cm). Out of 15,345 children with a known primary diagnosis, 3,981 (25.9%) were diagnosed with severe or cerebral malaria; 2,409 (15.7%) with malaria but not severe or cerebral; 3,000 (19.6%) with severe pneumonia; 920 (6%) with pneumonia but not severe; 1,798 (11.7%) with sepsis or septicemia; 23 (0.1%) with meningitis including bacterial, viral, cryptococcal or unspecified form; and 2,395 (11.6%) had other diagnoses.

**Table 1:** Baseline characteristics of study participants.

Characteristic	District hospital				
	Ntchisi	Mchinji	Balaka	Machinga	Total
	(*n* = 5,072)	(*n* = 4,225)	(*n* = 2,623)	(*n* = 3,543)	(*N* = 15,463)
Hospitalization outcome, *n* (%)					
Discharged alive	4,802 (96.2)	3,955 (94.0)	2,478 (94.9)	3,363 (95.1)	14,598 (95.1)
Dead	75 (1.5)	100 (2.4)	75 (2.9)	90 (2.5)	340 (2.2)
Referred to other hospitals	13 (0.3)	33 (0.8)	10 (0.4)	22 (0.6)	78 (0.5)
Absconded	102 (2.0)	118 (1.8)	47 (1.8)	62 (1.8)	329 (2.1)
Age of child, *n* (%)					
1–11 months	1,233 (24.3)	922 (21.8)	576 (22.0)	795 (22.5)	3,526 (22.8)
12–35 months	2,902 (57.3)	2,470 (58.5)	1,481 (56.6)	2,002 (56.6)	8,855 (57.3)
36–59 months	932 (18.4)	830 (19.7)	562 (21.5)	741 (20.9)	741 (20.9)
Gender, Male, *n* (%)	2,889	2,268	1,463	1,987	8,607
	(57.0)	(53.7)	(55.8)	(56.1)	(55.7)
Fever on admission, > 37.5 °C, *n* (%)	2,534/4,569 (55.5)	1,495/3,543 (42.2)	448/729 (61.5)	1,337/2,303 (58.1)	5,814/11,144 (52.2)
History of fever in last 7 days, *n* (%)	4,367	3,429	2,181	2,947	12,924
	(86.1)	(81.2)	(83.2)	(83.2)	(83.6)
HIV status, *n* (%)					
Negative	912	2,683	2,067	1,992	7,654
	(18.2)	(63.6)	(79.2)	(56.3)	(49.9)
Positive	5 (0.1)	27 (0.6)	22 (0.8)	35 (1.0)	89 (0.6)
Exposed but uninfected^b^	6 (0.1)	42 (1.0)	75 (2.9)	24 (0.7)	147 (1.0)
Unknown	4,079	1,454	446	1,486	7,465
	(81.6)	(34.6)	(17.1)	(42.0)	(48.6)
Malaria positive by mRDT, *n* (%)	2,103/4,005	2,010/2,998	974/1,468	1,866/3,063	6,953/11,534
	(52.5)	(67.0)	(66.4)	(60.9)	(60.3)
Malnourished, MUAC^a^ <11.5 cm, *n* (%)	300 (5.9)	209 (4.9)	128 (4.9)	299 (8.4)	936 (6.1)
Severe anemia (HB < 5 g/dl), *n* (%)	211/2,051 (10.3)	285/2,046 (13.9)	142/1,628 (8.7)	248/2,635 (9.4)	886/8,360 (10.6)
Oxygen saturation, percentage, median (IQR)	98 (96–100)	96 (94–98)	95.5 (90–98)	100 (98–100)	98 (95–99)
Blantyre coma score, median (range)	5 (3–5)	5 (2–5)	5 (2–5)	5 (2–5)	5 (2–5)
Primary diagnosis, *n* (%)					
Malaria (not severe or cerebral)	392/4,992	866/4,206	449/2,610	702/3,537	2,409/15,345
	(7.9)	(20.6)	(17.2)	(19.9)	(15.7)
Severe or cerebral malaria	1,446/4,992	1,030/4,206	634/2,610	871/3,537	3,981/15,345
	(29.0)	(24.5)	(24.3)	(24.6)	(25.9)
Pneumonia (not severe)	396/4,992	201/4,206	189/2,610	134/3,537	920/15,345
	(7.9)	(4.8)	(7.2)	(3.8)	(6.0)
Severe pneumonia	1,279/4,992	566/4,206	384/2,610	771/3,537	3,000/15,345
	(25.6)	(13.5)	(14.7)	(21.8)	(19.6)
Sepsis or septicemia	557/4,992	551/4,206	418/2,610	272/3,537	1,798/15,345
	(11.2)	(13.1)	(16.0)	(7.7)	(11.7)
Severe anemia malaria	148/4,992	64/4,206	213/2,610	394/3,537	819/15,345
	(3.0)	(1.5)	(8.2)	(11.1)	(5.3)
Meningitis	14/4,992	3/4,206	3/2,610	3/3,537	23/15,345
	(0.3)	(0.1)	(0.1)	(0.1)	(0.1)
Other	760/4,992	925/4,206	320/2,610	390/3,537	2,395/15,345
	(15.2)	(22.0)	(12.3)	(11.0)	(11.6)

^a^ MUAC, mid upper arm circumference.

^b^ Children exposed to HIV-positive mothers.

Out of all 15,463 children hospitalized, 15,345 (99.2%) children had known hospitalization outcomes by the time the data was locked for the current analyses (Table 1). Among the 15,345 children with known outcomes, 14,598 (95.1%) were discharged alive, 340 (2.2%) were in-hospital deaths, 329 (2.1%) absconded from the hospital, and 78 (0.5%) were referred, mostly to central hospitals.

## Time to discharge

**Figure 2:**
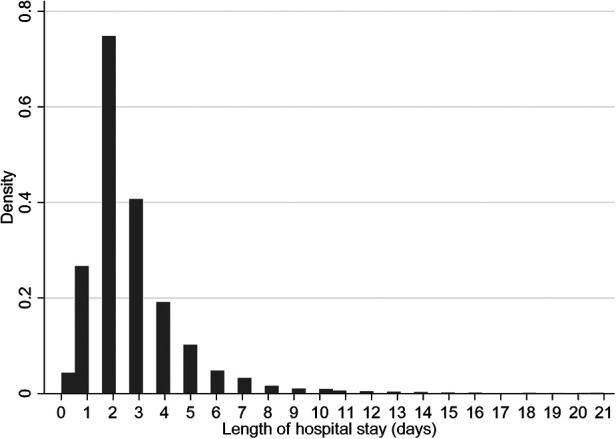
Distribution of length of stay (LOS) in days.

The median time to discharge was 2 days (IQR: 2–3 days) (Figure 2), with 6,091 (41.7%) of 14,598 children discharged by the second day of hospitalization. Most discharges occurred on day 2 and decreased exponentially to day 21 (Figure 2). The adjusted rate of discharge was marginally higher with 4% among children aged 36–59 months compared to children aged 1–11 months (cause-specific hazard ratio [CSHR] 1.04; [95% Confidence Interval (CI): 1.02–1.19]; *P* = 0.05) (Table 2). It was lower among children with HIV-positive status compared to children who were HIV-negative [CSHR: 0.59; (95% CI: 0.43–0.82); *P* = 0.002]. Compared to children with malaria (excluding severe and cerebral) as the primary diagnosis, children diagnosed with severe anemia malaria and meningitis had lower rates of discharge ([CSHR: 0.55; (95% CI: 0.47–0.65); *P* < 0.001] and [CSHR: 0.19; (95% CI: 0.08–0.47); *P* < 0.001], respectively). The rate of discharge increased by 26% for each 1-unit increase in BCS [CSHR: 1.26; (95% CI: 1.18–1.33); *P* < 0.001]. It also increased by 7% with each 1% increase in oxygen saturation [CSHR: 1.07; (95% CI: 1.05–1.11); *P* < 0.001]. The rate of discharge was lower among malnourished children [mid-upper arm circumference (MUAC) < 11.5 cm], compared to children who were not malnourished [CSHR: 0.66; (95% CI: 0.58–0.75); *P* < 0.001] (Table 2).

**Table 2:** Multivariable analysis for time to discharge, using a conventional Cox regression model to obtain a cause-specific hazard ratio, and the Fine-Gray competing risk method to obtain a sub-distribution-hazard ratio.

	Cause-specific hazard ratio (Rate of discharge)		Sub-distribution-hazard ratio (Association with cumulative incidence of discharge)	
Variable	CSH Ratio	*P* Value	SDH Ratio	*P* Value
Hospital				
Ntchisi	(*ref)*	-	-	-
Mchinji	0.98 (0.69–1.07)	0.7	0.94 (0.88–1.01)	0.05
Balaka	1.05 (0.97–1.15)	0.22	1.02 (0.95–1.09)	0.64
Machinga	1.26 (1.17–1.36)	**<0** **.** **001**	1.22 (1.14–1.29)	**<0** **.** **001**
Age of child				
1–11 months	(*ref)*	*-*	*-*	*-*
12–35 months	1.06 (0.99–1.14)	0.11	1.08 (1.02–1.15)	**0** **.** **006**
36–59 months	1.04 (1.01–1.19)	0.05	1.10 (1.02–1.18)	**0** **.** **009**
Gender				
Female	(*ref)*	*-*	*-*	*-*
Male	0.98 (0.94–1.04)	0.61	0.99 (0.95–1.03)	0.57
History of fever in last 7 days				
No	(*ref)*	* *	* *	
Yes	0.98 (0.94–1.11)	0.24	0.95 (0.87–1.15)	0.16
HIV status				
Negative	(*ref)*	-	-	
Positive	0.59 (0.43–0.82)	**0** **.** **002**	0.60 (0.46–0.76)	**<0** **.** **001**
Exposed	0.92 (0.69–1.22)	0.56	0.83 (0.65–1.08)	0.17
Unknown	1.03 (0.97–1.09)	0.38	0.97 (0.93–1.02)	0.27
Primary diagnosis				
Malaria^a^	(*ref)*	-	-	-
Severe or cerebral malaria	0.97 (0.88–1.06)	0.51	0.94 (0.86–0.97)	**0** **.** **04**
Pneumonia (excluding severe)	1.06 (0.92–1.22)	0.44	1.03 (0.92–1.14)	0.62
Severe pneumonia	0.92 (0.83–1.01)	0.11	0.93 (0.85–1.01)	0.071
Sepsis or septicemia	0.92 (0.82–1.02)	0.13	0.90 (0.82–0.98)	**0** **.** **027**
Severe anemia malaria	0.55 (0.47–0.65)	**<0** **.** **001**	0.54 (0.48–0.61)	**<0** **.** **001**
Meningitis	0.19 (0.08–0.47)	**<0** **.** **001**	0.18 (0.09–0.37)	**<0** **.** **001**
Other	0.62 (0.54–0.69)	**<0** **.** **001**	0.62 (0.56–0.68)	**<0** **.** **001**
Blantyre coma score, per 1 unit increase	1.26 (1.18–1.33)	**<0** **.** **001**	1.29 (1.23–1.37)	**<0** **.** **001**
Oxygen saturation, per 1% increase	1.02 (1.01–1.03)	**<0** **.** **001**	1.01 (1.01–1.02)	**<0** **.** **001**
Malnourished				
No	(*ref)*	-	-	-
Yes	0.66 (0.58–0.75)	**<0** **.** **001**	0.61 (0.55–0.68)	**<0** **.** **001**

The bold values are those that are significant results (i.e., *P* < 0.05).

CSH, cause-specific hazard; SDH, sub-distribution hazard.

^a^ Excluding severe or cerebral.

The cumulative incidence of discharge was 8% and 10% higher among children aged 12–35 months and 36–59 months, respectively, compared to children aged 1–11 months (sub-distribution-hazard ratio [SDHR] 1.04; [95% CI: 1.02–1.19]; *P* = 0.05) (Table 2). It was 40% lower among children with HIV-positive status compared to children who were HIV-negative [SDHR: 0.60; (95% CI: 0.46–0.76); *P* < 0.001]. Compared to children with malaria (excluding severe and cerebral) as the final primary diagnosis, the cumulative incidence of discharge was lower among children diagnosed with severe and cerebral malaria [SDHR: 0.94; (95% CI: 0.86–0.97); *P* = 0.04], sepsis or septicemia [SDHR: 0.90; (95% CI: 0.82–0.98); *P* = 0.027], severe anemia malaria [SDHR: 0.54; (95% CI: 0.48–0.61); *P* < 0.001], and meningitis [SDHR: 0.18; (95% CI: 0.09–0.37); *P* < 0.001]. The cumulative incidence of discharge increased by 29% for each unit increase in BCS [SDHR: 1.29; (95% CI: 1.23–1.37); *P* < 0.001], and it was 39% lower among malnourished children compared to children who were not malnourished [SDHR: 0.61; (95% CI: 0.55–0.68); *P* < 0.001].

**Figure 3:**
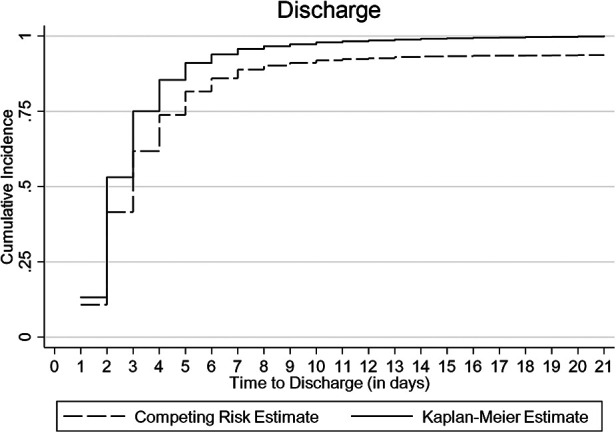
Cumulative incidence of discharge estimated using Kaplan-Meier (K-M) and competing risk methods accounting for death, referral, and abscondment.

**Figure 4:**
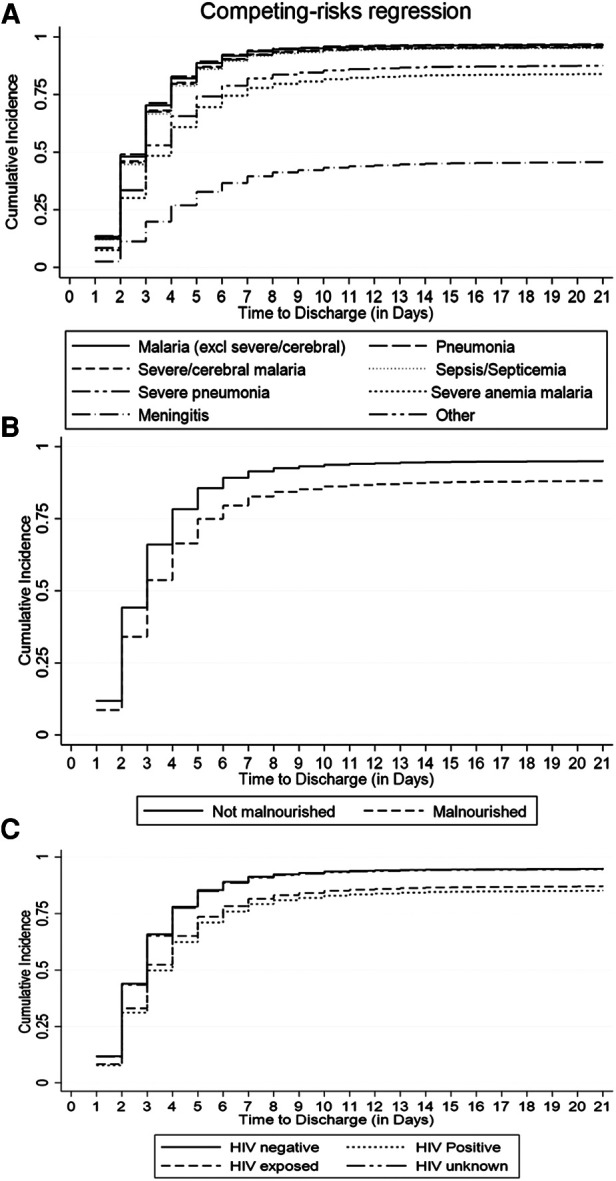
Cumulative incidence of discharge estimated using the Fine-Gray competing risk method accounting for death, abscondment, and referral, disaggregated by (**A**) primary diagnosis, (**B**) nutritional status, and (**C**) HIV status.

The cumulative incidence of discharge was overestimated to 100% by the conventional K-M complement compared with 80% by the Fine-Gray competing risk model after accounting for the effect of death, referral, and abscondment (Figure 3). Median time to discharge for children diagnosed with malaria (including severe and cerebral) or pneumonia (including severe pneumonia) was 2 (IQR: 2–3) days, 3 (IQR: 2–5) days for severe anemia malaria, and 6 (IQR: 4–14) days for those diagnosed with meningitis, (Figure 4A). Among children who were malnourished, the median time to discharge was 3 (IQR: 2–5) days, but it was 2 (IQR: 1–3) days for those who were not malnourished (Figure 4B). The median time to discharge was 2 (IQR: 2–3) days for children with negative or unknown HIV status and 4 (IQR: 2–6) days for children who were HIV-positive (Figure 4C).

## Discussion

LOS is critical in planning healthcare delivery and interventions that seek to reduce it prove effective in lowering health-related expenditures (3, 4, 23) and minimizing chances of in-hospital acquired infections (5). Using a competing risk approach, this study has demonstrated that children in similar settings can be risk-stratified based on LOS (i.e., time to discharge) in order to enhance patient care while accounting for death, referral, and abscondment to produce estimates relevant to the real-world (7). Factors for risk-stratifying patients identified in this study include the child's age, type of diagnosis (disease), nutritional status, and HIV status.

Among under-five children hospitalized in similar settings to this study, a median LOS (time to discharge or in-hospital death, referral, or abscondment) can be expected to be 2 days. Based on the pattern of cumulative incidence of discharge, hospital leadership and planners can expect most children diagnosed with malaria (excluding severe or cerebral) to be discharged within the first 4 days, cerebral or severe malaria and severe anemia malaria to be discharged within the first week, and children with meningitis or sepsis within the first 2 weeks (Figure 4A). Knowledge of projected discharge times is essential for timely resource allocation including laboratory and pharmacy stocks and bed availability (19, 24). Public health planning in healthcare delivery should consider allocating more resources to strategies that promote prevention of diseases that drain much-needed resources as a result of longer LOS.

Malnourished children had a 39% lower cumulative incidence of discharge when compared to children who were well-nourished (Table 2, Figure 4B). Malnutrition at admission has also been associated with prolonged length of hospital stay in similar settings such as a cohort study in Ethiopia (25) and a cross-sectional study in Cameroon (26). Therefore, from a clinical care perspective, it is essential to assess the nutritional status of patients early in admission and to institute appropriate nutritional therapy. Nutritional support therapy has the potential to reduce the cost of healthcare spending related to hospital-acquired conditions (27). Furthermore, community-based nutritional support programs should also consider targeting children under 1 year of age, and those that are infected with HIV. This study has demonstrated that the cumulative incidence of discharge among children aged 1 year or above was at least 8% higher compared to children under 1 year and that it was 40% lower among children with HIV-positive status compared to children who were HIV-negative.

This study has demonstrated the advantage of using a competing risk approach to estimate the cumulative incidence of discharge (sub-distribution hazard ratio) accounting for competing events (death, referral, and abscondment) over the conventional Cox proportional hazards regression (cause-specific hazard ratio). For example, the competing risk approach revealed that children who were younger and those diagnosed with severe or cerebral malaria or sepsis had reduced the cumulative incidence of discharge yet these were non-significant in the CSH model for discharge (Table 2). Therefore, using the conventional Cox regression and the K-M alone overestimated the cumulative incidence of discharge, the contribution of time to death, referral, or abscondment on resource use would be overlooked and consequently, the aforementioned factors would not have been considered in service planning. Researchers should understand that competing events affect the cumulative probability of cause-specific events and therefore use competing risk methods such as the Fine-Gray model to correctly estimate cumulative incidences (10, 13, 20, 28). Furthermore, the cumulative incidence is recommended for informing health care planning as it describes the trend of discharge over time rather than the rate of discharge alone (18, 19).

The strengths of this study include the prospective nature of the data collected over a period of about 20 months and from hospitals whose catchments cover all socio-demographics of the population and therefore are nationally representative. Secondly, the level of data missing in this study was minimal. The main limitation of the study is that the unique identity number was not maintained for children who had repeated hospitalization for the same or different disease conditions. We attempted to identify repeated hospitalizations by matching key variables such as age, gender, and location and only 3% of all records were identified as repeated hospitalizations. Further, this study did not assess the fitness of the Fine-Gray model. We recommend further research to consider conducting simulation studies with a more methodological focus as a way of assessing assumptions under different conditions of competing risk data in similar settings.

## Conclusion

In conclusion, this study has presented a useful statistical method for modeling the length of stay as the time to discharge where there are significant rates of in-unit mortality, referrals, and abscondment. In similar settings to this study, patient care in under-five children can be enhanced by risk-stratifying by LOS based on their age, HIV status, diagnosis, and nutritional status. In healthcare systems that are increasingly focusing on costs and resource planning, it is critical to consider not only the LOS for children discharged alive but also for those who die in hospital, get referred to other hospitals, or abscond before discharge.

## Data Availability

The raw data supporting the conclusions of this article will be made available by the authors, without undue reservation.
